# Supplementary material for: DNA translocation mechanism of an XPD family helicase
Source: eLife. 2018 Dec 6;7:e42400. doi: 10.7554/eLife.42400 (PMC6300356; doi:10.7554/eLife.42400)
Supplement: Supplementary file 1. [file elife-42400-supp1.docx]

**Table S1 Primers used for cloning and mutagenesis.**

| **Primers** | **Sequence (5′→3′)** |
| --- | --- |
| DinG_F | ttttttcatatggcattaaccgccgcgct |
| DinG_R | tttggatccttaacgccgccgacggcgtg |
| R117AF | gcggacaaacgtaggccccacgcccaaaag |
| R117AR | cttttgggcgtggggcctacgtttgtccgc |
| R211AF | tgaatctcccgagcagcgacaaaaaacgggcattcac |
| R211AR | gtgaatgcccgttttttgtcgctgctcgggagattca |
| F615AF | gtcgatgggcggagcagcgattttgtggatatgcacctgg |
| F615AR | ccaggtgcatatccacaaaatcgctgctccgcccatcgac |
| I618AF | caccgggctgtcggcgggcggaaaagcg |
| I618AR | cgcttttccgcccgccgacagcccggtg |
| Y636AF | ctttgcacctcaaacggagcgcggttgaggcttttcag |
| Y636AR | ctgaaaagcctcaaccgcgctccgtttgaggtgcaaag |
| F638AF | caggctttgcacctcagccggatagcggttgagg |
| F638AR | cctcaaccgctatccggctgaggtgcaaagcctg |
| R673AF | ttttggtcagcaaggctttgtcgtagataaccacttcgccc |
| R673AR | gggcgaagtggttatctacgacaaagccttgctgaccaaaa |
